# Overexpression of DGKI in Gastric Cancer Predicts Poor Prognosis

**DOI:** 10.3389/fmed.2020.00320

**Published:** 2020-07-07

**Authors:** Chao Huang, Jiefeng Zhao, Chen Luo, Zhengming Zhu

**Affiliations:** Department of Gastrointestinal Surgery, The Second Affiliated Hospital of Nanchang University, Nanchang, China

**Keywords:** gastric cancer, DGKI, TCGA, prognosis, GSEA

## Abstract

**Background:** Diacylglycerol kinase iota (DGKI) is overexpressed in a variety of cancers and is associated with poor prognosis in colon cancer. This study evaluated the prognostic value of DGKI in gastric cancer (GC) using data from The Cancer Genome Atlas (TCGA).

**Methods:** RNA sequencing results and clinical data of gastric adenoma and adenocarcinoma samples were obtained from the TCGA database (https://portal.gdc.cancer.gov). The Wilcoxon or Kruskal–Wallis test and logistic regression were used to analyze the relationship between DGKI and the clinicopathological characteristics of GC patients. Univariate Cox regression and Kaplan-Meier analysis were used to analyze the clinicopathological characteristics of GC patients and the relationship between DGKI and overall survival time, and multivariate Cox regression analysis was used to identify independent risk factors affecting the prognosis of GC patients. Gene set enrichment analysis (GSEA) was performed using the TCGA dataset.

**Results:** DGKI was overexpressed in gastric tumors and was related to poor prognosis (*p* = 0.003). Overexpression of DGKI in GC was significantly correlated with high grade (OR = 1.71 for G3 vs. G2), stage (OR = 2.08 for II vs. I) and T classification (OR = 4.64 for T4 vs. T1; OR = 3.99 for T3 vs. T1; OR = 3.37 for T2 vs. T1) (all *p* <0.05). DGKI (OR = 7.34; *p* = 0.000) was an independent risk factor affecting the survival of GC patients. The MAPK signaling pathway was differentially enriched with DGKI overexpression.

**Conclusion:** DGKI overexpression may be a potential molecular marker for poor prognosis in GC. The MAPK signaling pathway may be one of the key pathways related to DGKI regulation in GC.

## Introduction

Gastric cancer (GC) is a common malignant tumor of the digestive system, and its mortality rate ranks third (1). After radical gastrectomy, the 5-year survival rate of early gastric cancer can reach more than 90% (2, 3), but that of advanced gastric cancer is <10% (4). Postoperative recurrence is the main cause of the short survival time in patients with GC. Adjuvant chemotherapy can prevent recurrence and significantly improve the survival rate (5). Therefore, early identification and adjuvant chemotherapy are crucial for improving the survival of patients with advanced gastric cancer. Metabolic reprogramming has become a new marker of cancer development, and its success has confirmed that cancer is a metabolic disease (6). Recent studies have found that mitochondrial dysfunction, signaling, fatty acid metabolism, and mitochondrial autophagy are also related to tumor growth (7, 8). Diacylglycerol kinase iota (DGKI) is a member of the type IV diacylglycerol kinase subfamily. Diacylglycerol kinases regulate the intracellular concentration of diacylglycerol by phosphorylating, producing phosphatidic acid (PA) (9). Studies have shown that PA can regulate some signaling proteins, including protein kinases and phosphatases, and can mediate growth factors to induce mitosis of cells (10). The Mitogen-activated protein kinase (MAPK) signaling pathway is an important pathway for extracellular signals regulating cell mitosis, and Raf protein transfer from the cytoplasm to the membrane and activation by Ras or other kinases are key steps in the activation of this signaling pathway (11). Moreover, studies have indicated that Raf can directly bind to PA (12), so an increase in PA content promotes the translocation of the Raf protein to the cell membrane and activates the MAPK signaling pathway. Recently, studies (13) showed that DGKI was overexpressed in a variety of cancers and was associated with poor prognosis in colon cancer. However, a correlation between DGKI and the prognosis of GC has not been reported. Therefore, the purpose of this study was to evaluate the prognostic value of DGKI expression in GC based on data obtained from The Cancer Genome Atlas (TCGA) and to conduct gene set enrichment analysis (GSEA) to identify the signaling pathways related to DGKI regulation in GC.

## Materials and Methods

### Data Extraction and Processing

RNA sequencing results from 373 tissues to 348 human gastric adenoma and adenocarcinoma samples were obtained from the TCGA database (https://portal.gdc.cancer.gov). A Perl language (http://www.perl.org/) script was used to combine the RNA-seq results of 30 normal samples and 343 cancer samples into a single matrix file. The Ensembl database (http://www.ensembl.org/index.html) and a Perl script were then used to convert the Ensembl ID in the matrix file to the gene name. In addition, 406 sets of clinical data were downloaded, and then Perl language scripts were used to organize and extract relevant clinical data.

### GSEA

GSEA is used to assess whether a predefined set of genes show statistically significant, concordant differences between two biological states (14). To identify the potential mechanism by which DGKI expression affects the prognosis of gastric cancer patients, GSEA was used to first generate an ordered gene list based on the correlation between all genes and DGKI expression. GSEA was then used to elucidate the significant survival differences observed between the high and low DGKI groups. Gene set permutations were conducted 1,000 times for each analysis, and the expression level of DGKI was used as the phenotypic label. Gene sets with normalized (NOM) *p* <0.05 and false discovery rates (FDRs) <0.05 were defined as significantly enriched.

### Statistical Analysis

The Wilcoxon test was used to analyze the expression level of DGKI in tumor samples and normal samples. Kaplan-Meier analysis was used to compare the survival times between the DGKI high expression group and the DGKI low expression group, and the *p*-value was calculated by a log-rank test. The cut-off value for DGKI expression was determined by its median value. The Wilcoxon or Kruskal–Wallis test and logistic regression were used to analyze the relationship between DGKI and clinicopathological characteristics. Univariate Cox regression analysis was used to analyze the clinicopathological characteristics of GC patients and the relationship between DGKI and overall survival time. The factors affecting the survival of GC patients in univariate Cox regression analysis were analyzed by multivariate Cox regression to find independent risk factors affecting the prognosis of GC patients. A *p* <0.05 was considered statistically significant. All statistical analyses were performed using R (version x64 3.5.1).

## Results

### Clinical Characteristics of Patients

We identified some genes that were related to the prognosis of GC patients and can be used as independent risk factors affecting the prognosis of GC patients in the data downloaded from the TCGA database (Supplementary Tables 1, 2). Next, we further analyzed DGKI. The clinical data of 406 patients were downloaded from the TCGA database, and the patient's age, sex, histological grade, stage, TNM stage, survival time, and survival status were extracted. After deleting samples with incomplete clinical data, a total of 292 patients were obtained for further analysis (Table 1).

**Table 1:** Clinical characteristics of patients with gastric cancer.

	***n* (%)**
**Age**
≤65 years	129 (44.18)
>65 years	163 (55.82)
**Sex**
Male	176 (60.27)
Female	116 (39.73)
**Histological grade**
G1	5 (1.71)
G2	101 (34.59)
G3	186 (63.70)
**Stage**
I	41 (14.04)
II	94 (32.19)
III	126 (43.15)
IV	31 (10.62)
**T classification**
T1	15 (5.14)
T2	61 (20.89)
T3	142 (48.63)
T4	74 (25.34)
**M classification**
M0	273 (93.49)
M1	19 (6.51)
**N classification**
N0	92 (31.51)
N1	76 (26.03)
N2	65 (22.26)
N3	59 (20.20)
**Survival status**
Death	102 (34.93)
Survival	190 (65.07)

### Relationship Between the Expression of DGKI and Survival

**Figure 1:**
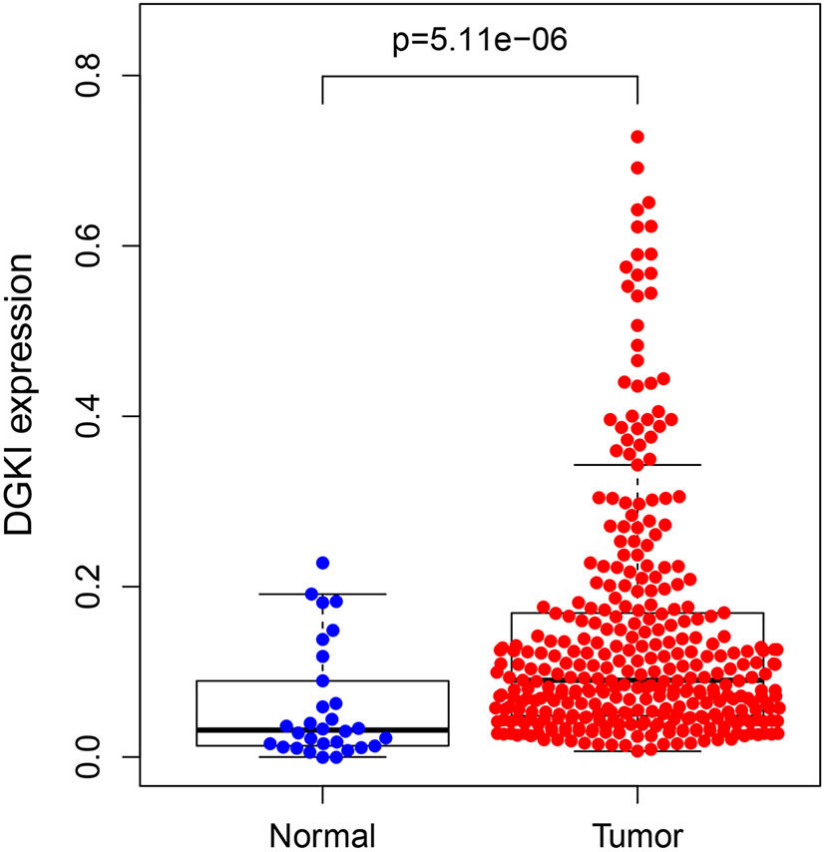
Expression level of DGKI in gastric tumors and normal tissues.

**Figure 2:**
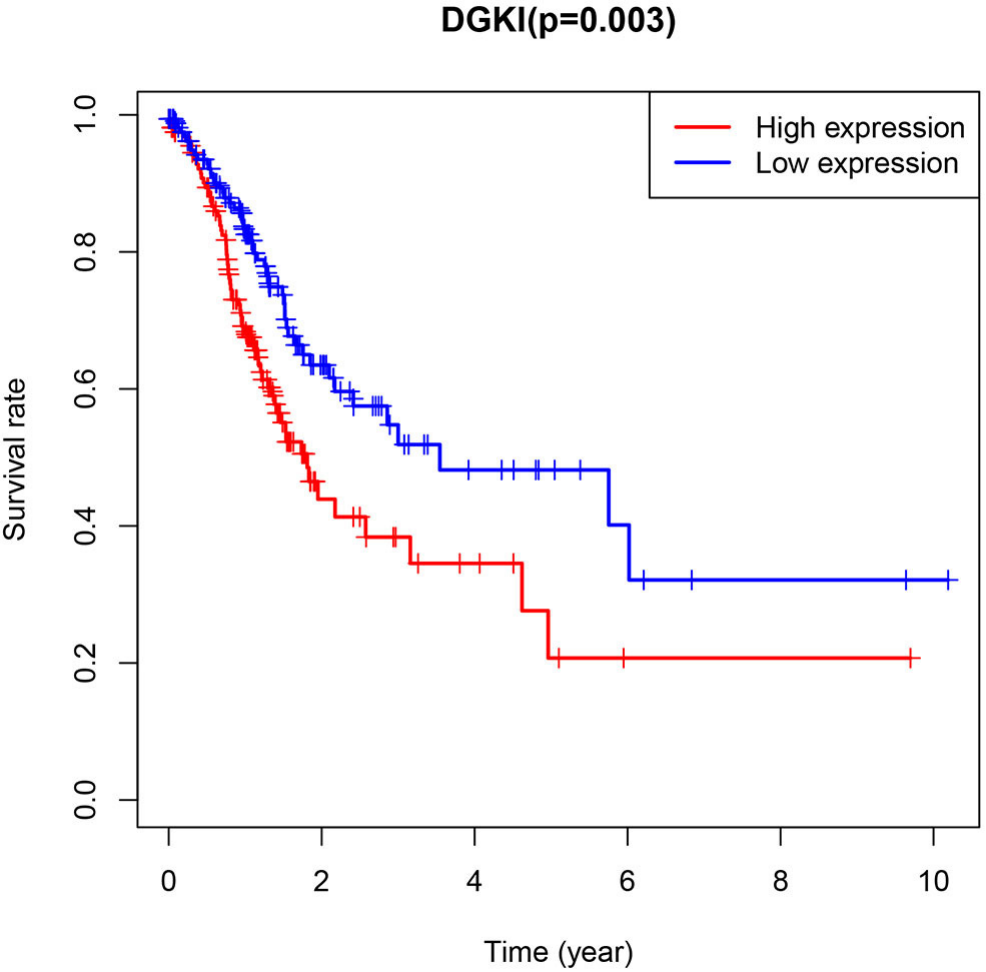
Impact of DGKI expression on overall survival in gastric cancer patients.

The difference in expression of DGKI between gastric tumors and normal tissues was statistically significant (*p* <0.001), indicating that DGKI was overexpressed in gastric tumors (Figure 1). The difference in survival time between patients with high expression of DGKI and those with low expression of DGKI was statistically significant (*p* = 0.003), showing that GC patients with high expression of DGKI had poor prognosis (Figure 2).

### Association Between DGKI Expression and Clinical Characteristics

**Figure 3:**
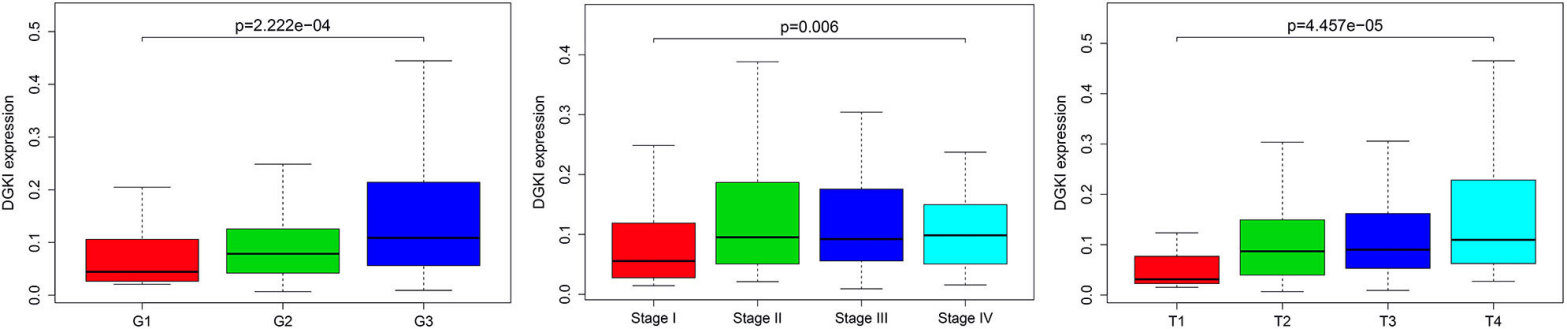
Association between DGKI expression and clinicopathologic characteristics, including grade, stage, and T classification.

We analyzed 292 GC samples with DGKI expression and acquired clinical data. Histological grade (*p* <0.001), stage (*p* = 0.006), and T classification (*p* <0.001) were related to the expression level of DGKI (Figure 3). Logistic regression analysis was performed after grouping according to the median expression of DGKI. The results showed that overexpression of DGKI in GC was significantly correlated with high grade (OR = 1.71 for G3 vs. G2), stage (OR = 2.08 for II vs. I) and T classification (OR = 4.64 for T4 vs. T1; OR = 3.99 for T3 vs. T1; OR = 3.37 for T2 vs. T1) (all *p* <0.05) (Table 2). These results suggest that GC patients with high DGKI expression are more likely to have advanced gastric cancer than GC patients with low DGKI expression.

**Table 2:** DGKI expression associated with clinical pathological characteristics (logistic regression).

**Clinical characteristics**	**Total (*n*)**	**Odds ratio in DGKI expression**	***p*-value**
Age (>65 years vs. ≤ 65 years)	341	0.96 (0.63–1.48)	0.865
Sex (male vs. female)	406	0.83(0.54–1.29)	0.410
Histological grade (G3 vs. G2)	328	1.71 (1.09–2.68)	0.019
Stage (IIvs. I)	152	2.08 (1.05–4.23)	0.039
**T classification**			
(T4 vs. T1)	104	4.64 (154–17.32)	0.011
(T3 vs. T1)	176	3.99 (1.38–14.50)	0.018
(T2 vs. T1)	93	3.37 (1.10–12.67)	0.046
M classification (M1 vs. M0)	327	0.92 (0.39–2.15)	0.841
**N classification**			
(N3 vs. N0)	167	1.62 (0.87–3.07)	0.132
(N2 vs. N0)	173	0.94 (0.51–1.72)	0.840
(N1 vs. N0)	191	0.97 (0.55–1.71)	0.907

### Univariate and Multivariate Cox Regression Analysis

**Figure 4:**
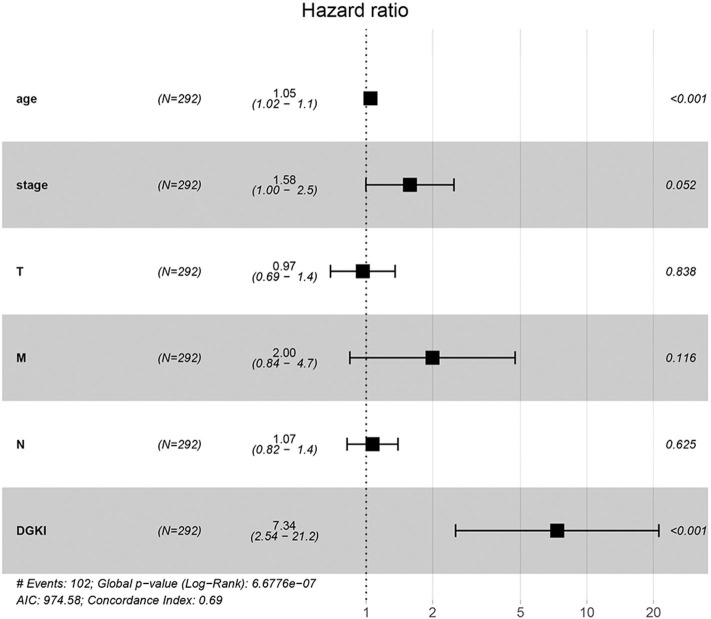
Forest plot of the correlation of DGKI expression with overall survival among patients with gastric cancer.

Univariate Cox regression analysis showed that the factors affecting the survival of GC patients were age (*p* = 0.005), stage (*p* = 0.000), T classification (*p* = 0.045), M classification (*p* = 0.017), N classification (*p* = 0.013), and DGKI (*p* = 0.015) (Table 3). Multivariate Cox regression analysis of the above factors found that age (OR = 1.05; *p* = 0.000) and DGKI (OR = 7.34; *p* = 0.000) were independent risk factors affecting the survival of patients with GC (Table 3 and Figure 4).

**Table 3:** Univariate analysis and multivariate analysis of the correlation of DGKI expression with overall survival among patients with gastric cancer.

**Clinicopathologic variable**	**Univariate analysis**		**Multivariate analysis**	
	**HR (95% CI)**	***p*-value**	**HR (95% CI)**	***p*-value**
Age	1.03 (1.01–1.05)	0.005	1.05 (1.02–1.07)	0.000
Sex	1.51 (0.99–2.31)	0.059		
Histological grade	1.24 (0.85–1.83)	0.269		
Stage	1.53 (1.21–1.94)	0.000	1.58 (1.0–2.50)	0.052
T classification	1.29 (1.01–1.64)	0.045	0.97 (0.69–1.35)	0.838
M classification	2.23 (1.16–4.30)	0.017	2.0 (0.84–4.73)	0.116
N classification	1.25 (1.05–1.50)	0.013	1.07 (0.82–1.39)	0.625
DGKI	3.29 (1.26–8.57)	0.015	7.34 (2.54–21.18)	0.000

### GSEA Identifies DGKI-Related Signaling Pathways

**Figure 5:**
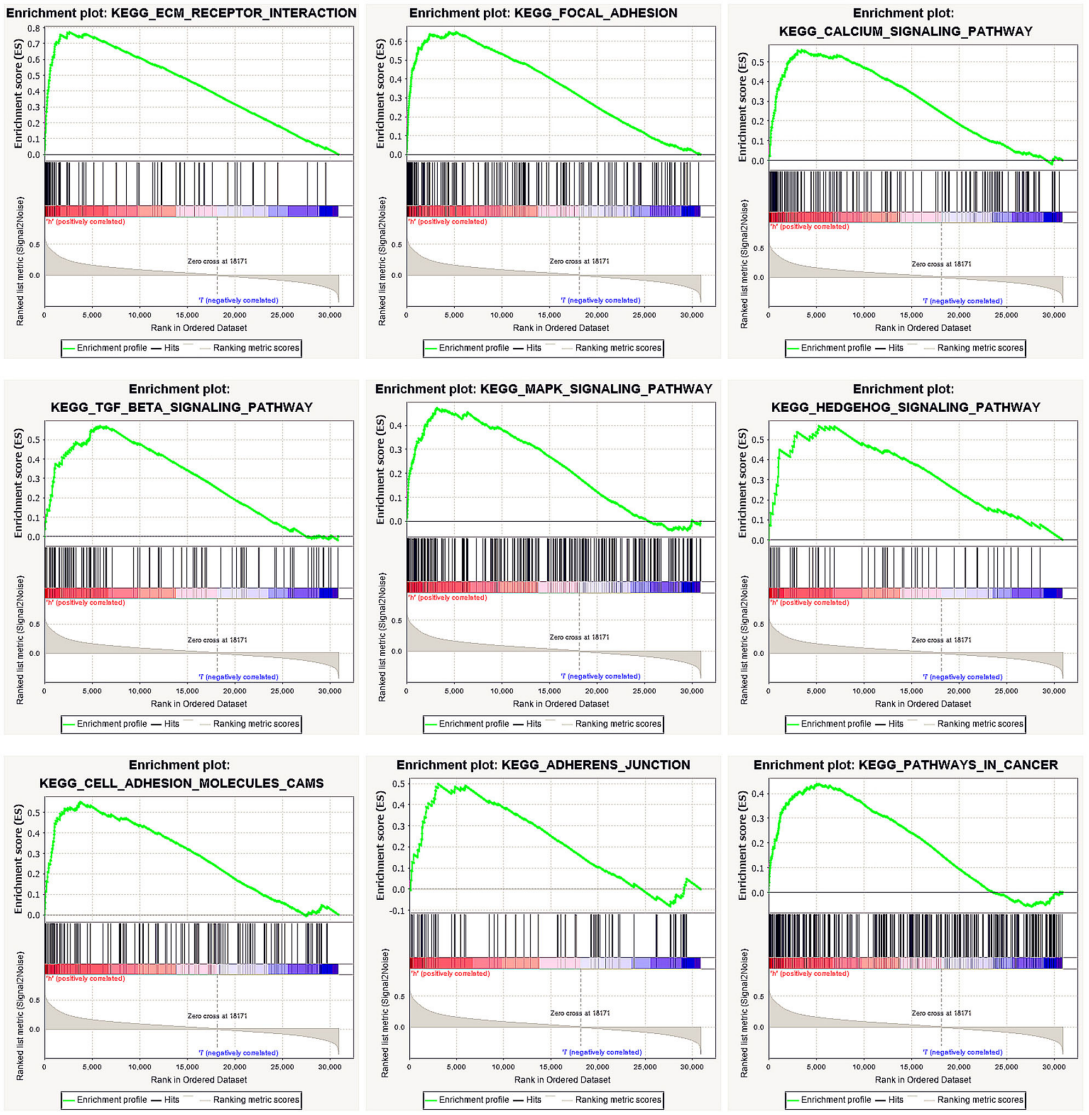
Enrichment plots from gene set enrichment analysis (GSEA). GSEA results showing differential enrichment of genes related to ECM receptor interaction, focal adhesion, calcium signaling pathway, TGF-beta signaling pathway, MAPK signaling pathway, Hedgehog signaling pathway, cell adhesion molecules (CAMs), adherens junction, and pathways in cancer in gastric cancer with high DGKI expression.

We performed GSEA of the high and low DGKI expression datasets to identify differentially activated signaling pathways in GC. GSEA was performed using the MSigDB collection (c2.cp.kegg.v7.0.symbols.gmt). We screened differentially enriched pathways associated with the DGKI high expression phenotype with FDR <0.05 and NOM *p* <0.05 cut-offs (Table 4). Pathways related to ECM receptor interactions, focal adhesion, calcium signaling, TGF-beta signaling, MAPK signaling, Hedgehog signaling, cell adhesion molecules (CAMs), adherens junctions, and cancer were differentially enriched with the DGKI high expression phenotype (Figure 5).

**Table 4:** Gene sets enriched in the high DGKI expression phenotype.

**Name**	**ES**	**NES**	**NOM *p*-val**	**FDR *q*-val**
KEGG_ECM_RECEPTOR_INTERACTION	0.773	2.344	0.000	0.000
KEGG_FOCAL_ADHESION	0.649	2.327	0.000	0.000
KEGG_CALCIUM_SIGNALING_PATHWAY	0.560	2.128	0.000	0.002
KEGG_TGF_BETA_SIGNALING_PATHWAY	0.570	2.033	0.000	0.006
KEGG_MAPK_SIGNALING_PATHWAY	0.472	1.936	0.000	0.016
KEGG_HEDGEHOG_SIGNALING_PATHWAY	0.568	1.864	0.008	0.031
KEGG_CELL_ADHESION_MOLECULES_CAMs	0.551	1.830	0.006	0.037
KEGG_ADHERENS_JUNCTION	0.498	1.820	0.006	0.037
KEGG_PATHWAYS_IN_CANCER	0.439	1.802	0.010	0.039

*NES, normalized enrichment score; NOM, nominal; FDR, false discovery rate. Gene sets with NOM p <0.05 and FDR q <0.05 are considered as significant*.

## Discussion

Metabolic reprogramming has become a new marker of cancer development and its success has confirmed that cancer is a metabolic disease (6). TP53 (tumor protein p53) is the most common mutated gene in human cancer, with more than half of tumors accompanied by TP53 changes (15). Studies have shown that TP 53 controls various metabolic pathways, including glycolysis, lipid metabolism and mitochondrial function (16, 17). DGKI regulates the concentration of intracellular diacylglycerin through phosphorylation to produce phosphatidic acid (9). Moreover, studies have found that PA can regulate some signaling proteins, including protein kinases and phosphatases, and it can mediate growth factors to induce mitosis in cells (10). Previous studies (9) showed that DGKI was expressed in the retina and brain, suggesting that it may play an important role in retinopathy. In addition, Ohanian and Ohanian (18) showed that DGKI plays a role in regulating the contraction of vascular smooth muscle which plays a role in hypertension. Recently, Etcheverry et al. (19) indicated that DGKI methylation status was associated with the prognosis of patients with glioblastoma. Penrose et al. (13) demonstrated that DGKI was overexpressed in a variety of cancers and was associated with poor prognosis of colon cancer. Our study found that DGKI was overexpressed in gastric tumors and was associated with a poor prognosis for patients. Meanwhile, overexpression of DGKI in GC was significantly correlated with high grade, stage and T classification. In addition, multivariate Cox analysis showed that DGKI was an independent risk factor affecting the survival of GC patients.

To further study the function of DGKI in GC, we performed GSEA. GSEA found that pathways related to ECM receptor interactions, focal adhesion, calcium signaling, TGF-beta signaling, MAPK signaling, Hedgehog signaling, cell adhesion molecules (CAMs), adherens junctions, and cancer were differentially enriched with the DGKI high expression phenotype. The MAPK pathways have different signaling cascades; the Ras-Raf-Mek-extracellular signal-regulated kinases 1 and 2 pathway, one of the MAPK pathways, is one of the most dysregulated pathways in human cancer and it regulates a variety of key cellular functions, including proliferation, growth, and aging (20). The Raf protein continuously activates the MAPK pathway, causing abnormal differentiation, proliferation and apoptosis, as well as the development of cancer (21). Ghosh et al. (12) showed that Raf can bind directly to PA, such that an increase in PA content promotes the translocation of the Raf protein to the cell membrane and activates the MAPK signaling pathway. We performed GSEA and found that the MAPK signaling pathway was differentially enriched with DGKI overexpression. However, the regulatory mechanism needs to be further elucidated.

In addition, several studies have indicated that the AMP-activated protein kinase (AMPK) and MAPK3/1 pathways may be biological predictors and beneficial targets for cancer treatment using metabolic alterations (22, 23). Kim et al. (24) reported that AMPK may inhibit the MAPK3/1 pathway; the inhibition of AMPK by expressing a dominant-negative form potentiates MAPK3/1 activation under glucose deprivation in colon cancer cells. However, Kim et al. (25) also showed that the effects of AMPK activation and the association between the AMPK and MAPK3/1 pathways need to be further elucidated to improve the treatment strategies for human cancer. We performed GSEA of the high and low DGKI expression datasets to identify differentially activated signaling pathways in GC, and did not find that the AMPK pathway was differentially enriched in the DGKI high expression phenotypes. Therefore, the correlation between DGKI and AMPK pathway needs to be further studied, which may also be a direction of our future research.

In conclusion, DGKI overexpression may be a potential molecular marker for poor prognosis in GC. The MAPK signaling pathway may be one of the key pathways related to DGKI regulation in GC. However, it is necessary to carry out further experimental verification to prove the biological function of DGKI.

## Data Availability Statement

The datasets generated for this study can be found in the https://portal.gdc.cancer.gov.

## Author Contributions

CH and ZZ: designed the study. CH and JZ: collected data. CH and CL: analyzed the data. CH: wrote the manuscript with contribution from all authors. ZZ: provided critical comments for this paper. All authors contributed to the article and approved the submitted version.

## Conflict of Interest

The authors declare that the research was conducted in the absence of any commercial or financial relationships that could be construed as a potential conflict of interest.
