# Supplementary material for: Overexpression of DGKI in Gastric Cancer Predicts Poor Prognosis
Source: Front Med (Lausanne). 2020 Jul 7;7:320. doi: 10.3389/fmed.2020.00320 (PMC7358307; doi:10.3389/fmed.2020.00320)
Supplement: Supplementary file 1 [file Table_1.doc]

| Genes | KM | HR | HR.95L | HR.95H | Cox Pvalue |
| --- | --- | --- | --- | --- | --- |
| RAI14 | 0.000326173 | 1.030922103 | 1.001891144 | 1.060794268 | 0.036653388 |
| ANTXR1 | 0.028305519 | 1.010241658 | 1.001429421 | 1.019131439 | 0.022636655 |
| RGS5 | 0.002361937 | 1.009904504 | 1.00118283 | 1.018702156 | 0.025941437 |
| NUAK1 | 0.001648497 | 1.109167902 | 1.011423687 | 1.21635814 | 0.027715128 |
| MATN3 | 0.000483428 | 1.057663131 | 1.018345632 | 1.098498648 | 0.003725341 |
| AC105935.2 | 0.046827625 | 0.304746708 | 0.128085427 | 0.72506731 | 0.0072115 |
| COL8A1 | 0.042601491 | 1.022338095 | 1.005049691 | 1.039923887 | 0.011123147 |
| PXDN | 0.047436802 | 1.036803351 | 1.011019721 | 1.06324453 | 0.004909221 |
| OLFML2B | 0.046344389 | 1.012856019 | 1.000122234 | 1.025751934 | 0.047827409 |
| LUM | 0.027051546 | 1.001393062 | 1.000325002 | 1.002462263 | 0.010564608 |
| RPL23AP59 | 0.002617606 | 2.3370074 | 1.223208826 | 4.464980528 | 0.010171875 |
| LINC00449 | 0.021857555 | 0.374752666 | 0.170394127 | 0.824204233 | 0.014656751 |
| CTHRC1 | 0.004485898 | 1.011662694 | 1.004264324 | 1.019115567 | 0.00195997 |
| CCNO | 0.005079563 | 0.924709022 | 0.86970168 | 0.983195497 | 0.01236439 |
| AC010719.1 | 0.004958349 | 0.750645992 | 0.609053053 | 0.925156523 | 0.00715836 |
| ELOVL4 | 0.031599557 | 1.10991471 | 1.021242462 | 1.206286175 | 0.01409781 |
| CDH11 | 0.019968473 | 1.049079933 | 1.014895546 | 1.084415742 | 0.004586299 |
| MYL4 | 0.021747864 | 1.876501012 | 1.061703797 | 3.316608696 | 0.030310074 |
| KCND2 | 0.008824407 | 1.871981679 | 1.155659812 | 3.032307058 | 0.010839033 |
| SNCG | 0.004949946 | 1.025889646 | 1.008326034 | 1.043759191 | 0.003719206 |
| COL12A1 | 0.015715898 | 1.007649589 | 1.000609324 | 1.014739389 | 0.033151498 |
| AC129507.1 | 0.036766818 | 5.392711234 | 2.067244053 | 14.06768321 | 0.000572255 |
| FNDC1 | 0.013026147 | 1.019124566 | 1.007909623 | 1.030464296 | 0.000792396 |
| COL4A1 | 0.044756744 | 1.003600168 | 1.000112786 | 1.00709971 | 0.043025143 |
| SLC22A17 | 0.010323652 | 1.057079637 | 1.010890373 | 1.105379365 | 0.014886788 |
| CDH6 | 0.002724612 | 1.298647381 | 1.109297889 | 1.520317524 | 0.00115412 |
| CNRIP1 | 0.010966945 | 1.114160403 | 1.022065548 | 1.214553613 | 0.014057614 |
| AC011466.4 | 0.029012187 | 0.144257794 | 0.023137563 | 0.899416741 | 0.038126478 |
| RF00604 | 0.017477326 | 0.837213483 | 0.705269807 | 0.993841519 | 0.042299294 |
| PNMA2 | 0.001603964 | 1.059348303 | 1.000418748 | 1.121749097 | 0.048347574 |
| AP003170.5 | 0.007424661 | 0.31514807 | 0.118875633 | 0.835480779 | 0.020270127 |
| COL4A5 | 0.001218154 | 1.038371775 | 1.000457444 | 1.077722944 | 0.047248911 |
| VCAN | 0.003543264 | 1.022448662 | 1.00820272 | 1.036895901 | 0.001928024 |
| RGS2 | 0.006030992 | 1.009434329 | 1.002695919 | 1.016218024 | 0.005999618 |
| ZFP36 | 0.041110153 | 1.001386312 | 1.00030865 | 1.002465135 | 0.011678914 |
| AL365356.5 | 0.004662678 | 1.09280613 | 1.043381116 | 1.144572409 | 0.000171047 |
| LINC02407 | 0.003476701 | 3.193502504 | 1.485634289 | 6.864716518 | 0.002941732 |
| NOX4 | 0.031177287 | 1.750839304 | 1.274836601 | 2.404573471 | 0.000540256 |
| INHBA | 0.030798458 | 1.029630002 | 1.010135787 | 1.049500429 | 0.002753256 |
| AL022316.1 | 0.00124609 | 0.686450027 | 0.518026499 | 0.909632308 | 0.008808298 |
| FBXL7 | 0.031452139 | 1.072650036 | 1.012089791 | 1.136834014 | 0.018017669 |
| AL592148.1 | 0.033091939 | 2.638525227 | 1.103405539 | 6.309389551 | 0.029169868 |
| AC005363.2 | 0.021227963 | 0.431461133 | 0.192261086 | 0.968259948 | 0.041532651 |
| MAB21L2 | 0.017790748 | 1.129417367 | 1.044077111 | 1.221733122 | 0.002397675 |
| LINC00412 | 0.0312769 | 0.246869779 | 0.077195911 | 0.789480778 | 0.018349301 |
| DCN | 0.025344536 | 1.005756118 | 1.000472725 | 1.011067412 | 0.032692978 |
| AC124319.1 | 0.011052421 | 0.764787166 | 0.6281027 | 0.9312162 | 0.007599738 |
| AC004466.3 | 0.025021974 | 1.963147509 | 1.091946471 | 3.529429549 | 0.024204287 |
| BX322635.1 | 0.003089933 | 5.398210946 | 2.212074345 | 13.17346385 | 0.000212079 |
| TCEAL7 | 0.000269958 | 1.116567031 | 1.026000724 | 1.215127733 | 0.010627827 |
| AC017076.1 | 0.020694697 | 3.001361974 | 1.305529794 | 6.900013876 | 0.009662566 |
| P4HA3 | 0.004098111 | 1.154655951 | 1.04572008 | 1.274940006 | 0.00445277 |
| LRRC45 | 0.003110115 | 0.932864622 | 0.883608545 | 0.984866442 | 0.012041178 |
| LOX | 0.009285843 | 1.028781708 | 1.009228032 | 1.048714235 | 0.003753541 |
| AC005546.1 | 0.03611452 | 0.69404741 | 0.49583132 | 0.971503387 | 0.033299549 |
| AC005586.1 | 0.00111288 | 0.770454773 | 0.638558037 | 0.929595311 | 0.006488529 |
| MIR3142HG | 0.013854531 | 0.783337253 | 0.65132517 | 0.942105849 | 0.00950557 |
| COL5A2 | 0.008529802 | 1.009670582 | 1.002922325 | 1.016464244 | 0.004910916 |
| AL139147.1 | 0.004408105 | 7.989273929 | 2.486204552 | 25.67306775 | 0.00048461 |
| GUCY1A2 | 0.022262323 | 1.498251705 | 1.096244168 | 2.047680833 | 0.011198111 |
| TMEM200A | 0.027434037 | 1.049540508 | 1.00448192 | 1.096620313 | 0.030795462 |
| LINC01235 | 0.00246085 | 1.245558179 | 1.03654658 | 1.49671535 | 0.019131466 |
| FGD6 | 0.042586966 | 1.071615703 | 1.004875558 | 1.142788484 | 0.035012941 |
| CFAP157 | 0.005461087 | 0.587929419 | 0.372500229 | 0.927948428 | 0.022541522 |
| ANKRD53 | 0.005298572 | 1.665521532 | 1.040915767 | 2.664924543 | 0.0334054 |
| SERPINE1 | 0.000597286 | 1.001939678 | 1.000652648 | 1.003228364 | 0.003128551 |
| GPX8 | 0.003136434 | 1.058085868 | 1.022468053 | 1.094944436 | 0.001230284 |
| RPP25 | 0.020691782 | 0.974355327 | 0.949867581 | 0.999474371 | 0.045451795 |
| TRAF2 | 0.030454838 | 0.930603914 | 0.870273687 | 0.99511643 | 0.035455419 |
| IFIT2 | 0.025235943 | 1.029341799 | 1.000289705 | 1.059237673 | 0.047726687 |
| AL033527.3 | 0.030599796 | 0.187954923 | 0.059486858 | 0.593863154 | 0.00440296 |
| MCEMP1 | 0.002415817 | 1.221278269 | 1.099060243 | 1.35708722 | 0.000202642 |
| MMP8 | 0.032839628 | 1.077454336 | 1.007216168 | 1.152590559 | 0.030081179 |
| LINC01537 | 0.001962037 | 3.300864903 | 1.130742688 | 9.635887302 | 0.028906193 |
| RPS14P4 | 0.007733262 | 0.731308542 | 0.54749878 | 0.976828082 | 0.034115699 |
| OACYLP | 0.007529601 | 3.3789542 | 1.400523076 | 8.152190909 | 0.006736766 |
| CACNA2D3 | 0.013439985 | 1.729181425 | 1.023049359 | 2.922701994 | 0.040849016 |
| PDE1B | 0.043879625 | 1.353497727 | 1.094551205 | 1.673705249 | 0.005208614 |
| SPARC | 0.011898137 | 1.001455981 | 1.000434918 | 1.002478087 | 0.005183392 |
| AP000695.1 | 0.008376619 | 1.336898807 | 1.116337236 | 1.601038074 | 0.001597814 |
| PTH1R | 0.021317865 | 1.49665923 | 1.062937285 | 2.107357492 | 0.020913007 |
| LGR6 | 0.01369727 | 0.96131454 | 0.925874904 | 0.998110697 | 0.03952887 |
| BICC1 | 0.016552448 | 1.07392158 | 1.016884291 | 1.134158105 | 0.010428545 |
| NUDT10 | 0.003007466 | 1.629777451 | 1.16712188 | 2.275833043 | 0.004142551 |
| PLXDC1 | 0.010114599 | 1.198687353 | 1.06518863 | 1.3489173 | 0.00262765 |
| RNU7-45P | 0.049725013 | 0.959578624 | 0.924055001 | 0.996467888 | 0.032048314 |
| RMI1 | 0.034466322 | 0.903650497 | 0.830465262 | 0.983285224 | 0.018716076 |
| DGKI | 0.003036284 | 3.267886331 | 1.317724753 | 8.104181885 | 0.010607589 |
| PLCL1 | 0.009012465 | 1.440327585 | 1.096223623 | 1.892445582 | 0.008804806 |
| CHAF1A | 0.000448744 | 0.903153227 | 0.847274263 | 0.962717489 | 0.001772276 |
| MIR4635 | 0.003700587 | 0.890040871 | 0.802656352 | 0.98693887 | 0.027152819 |
| FEN1 | 0.006165593 | 0.968248433 | 0.943509518 | 0.993636005 | 0.014548589 |
| AC005324.5 | 0.001715184 | 0.369273547 | 0.148682064 | 0.917144602 | 0.031848828 |

Supplementary Table 1 Genes related to the prognosis of GC patients
