# Supplementary material for: Overexpression of DGKI in Gastric Cancer Predicts Poor Prognosis
Source: Front Med (Lausanne). 2020 Jul 7;7:320. doi: 10.3389/fmed.2020.00320 (PMC7358307; doi:10.3389/fmed.2020.00320)
Supplement: Supplementary file 2 [file Table_2.doc]

| Genes | HR | HR.95L | HR.95H | P value |
| --- | --- | --- | --- | --- |
| RAI14 | 1.039686001 | 1.004577869 | 1.076021096 | 0.026380451 |
| ANTXR1 | 1.012037479 | 1.00195296 | 1.022223497 | 0.019190567 |
| RGS5 | 1.01185257 | 1.001816982 | 1.021988689 | 0.020508024 |
| NUAK1 | 1.124149513 | 1.012778067 | 1.247768062 | 0.027913826 |
| MATN3 | 1.08224704 | 1.040335972 | 1.125846541 | 8.77E-05 |
| AC105935.2 | 0.280276671 | 0.105975782 | 0.741254376 | 0.010366672 |
| COL8A1 | 1.032290714 | 1.012213016 | 1.052766662 | 0.001517616 |
| PXDN | 1.036163775 | 1.006796675 | 1.06638748 | 0.015447281 |
| LUM | 1.001836515 | 1.000413973 | 1.003261079 | 0.011378461 |
| RPL23AP59 | 2.56737158 | 1.229658603 | 5.360347021 | 0.012059683 |
| LINC00449 | 0.373411992 | 0.157350735 | 0.88615103 | 0.025477486 |
| CTHRC1 | 1.011231535 | 1.002638078 | 1.019898645 | 0.010317041 |
| CCNO | 0.895915558 | 0.83769337 | 0.958184361 | 0.00134637 |
| AC010719.1 | 0.721997951 | 0.569313816 | 0.915630407 | 0.007207947 |
| ELOVL4 | 1.140317789 | 1.047508788 | 1.241349643 | 0.002432832 |
| CDH11 | 1.063854239 | 1.02125503 | 1.108230371 | 0.002990759 |
| KCND2 | 2.48877693 | 1.321348836 | 4.687642232 | 0.004764018 |
| SNCG | 1.024549293 | 1.004396977 | 1.045105947 | 0.016719118 |
| AC129507.1 | 10.21391738 | 3.414747602 | 30.55104517 | 3.23E-05 |
| FNDC1 | 1.023807809 | 1.007571666 | 1.040305584 | 0.003916498 |
| SLC22A17 | 1.087541373 | 1.039621201 | 1.137670373 | 0.000262265 |
| CDH6 | 1.501141529 | 1.22051802 | 1.846286457 | 0.000119456 |
| CNRIP1 | 1.179601806 | 1.05711134 | 1.316285587 | 0.003148592 |
| RF00604 | 0.823951682 | 0.679600522 | 0.998963879 | 0.048779606 |
| AP003170.5 | 0.349987901 | 0.122504447 | 0.999894568 | 0.049976993 |
| COL4A5 | 1.059494428 | 1.021989893 | 1.098375289 | 0.001672979 |
| VCAN | 1.02462796 | 1.007046396 | 1.042516472 | 0.005867295 |
| RGS2 | 1.011705186 | 1.003236408 | 1.020245454 | 0.006660635 |
| ZFP36 | 1.001546713 | 1.000401195 | 1.002693542 | 0.008122517 |
| AL365356.5 | 1.092052993 | 1.037748004 | 1.149199743 | 0.000715016 |
| LINC02407 | 3.021854802 | 1.242579651 | 7.348910341 | 0.014729186 |
| NOX4 | 1.982975513 | 1.336240205 | 2.942728315 | 0.000675878 |
| AL022316.1 | 0.738166849 | 0.553181118 | 0.985012465 | 0.039154441 |
| FBXL7 | 1.139300622 | 1.06038642 | 1.224087638 | 0.000369542 |
| MAB21L2 | 1.180272494 | 1.092254674 | 1.275383108 | 2.77E-05 |
| LINC00412 | 0.151671722 | 0.039755835 | 0.578639866 | 0.005766546 |
| DCN | 1.009704075 | 1.002255487 | 1.01720802 | 0.010578294 |
| AC124319.1 | 0.782613092 | 0.62489599 | 0.980136313 | 0.032785859 |
| BX322635.1 | 4.582972783 | 1.715376653 | 12.244331 | 0.002395584 |
| TCEAL7 | 1.26250549 | 1.127299969 | 1.413927221 | 5.50E-05 |
| AC017076.1 | 3.347722888 | 1.229052926 | 9.118605305 | 0.01810943 |
| P4HA3 | 1.164168668 | 1.027784464 | 1.318650686 | 0.016800384 |
| AC005546.1 | 0.659243261 | 0.448211774 | 0.969634675 | 0.034293174 |
| AC005586.1 | 0.799780972 | 0.652866177 | 0.979756075 | 0.030970011 |
| MIR3142HG | 0.703643788 | 0.560143592 | 0.883906533 | 0.002524263 |
| AL139147.1 | 4.897644208 | 1.270146038 | 18.88516601 | 0.021041511 |
| GUCY1A2 | 1.710799674 | 1.174524221 | 2.49193288 | 0.005137674 |
| TMEM200A | 1.061756482 | 1.009699108 | 1.116497794 | 0.019476589 |
| CFAP157 | 0.583385347 | 0.350123808 | 0.972051755 | 0.038566669 |
| ANKRD53 | 2.377771209 | 1.400547359 | 4.036847369 | 0.00133966 |
| SERPINE1 | 1.002108969 | 1.000526222 | 1.003694219 | 0.008993837 |
| TRAF2 | 0.906213975 | 0.837606447 | 0.980441079 | 0.014217469 |
| AL033527.3 | 0.186347498 | 0.051064817 | 0.680025743 | 0.010964792 |
| MCEMP1 | 1.21937805 | 1.053854905 | 1.410898996 | 0.007705978 |
| MMP8 | 1.700378853 | 1.04475296 | 2.767437237 | 0.032668346 |
| LINC01537 | 5.909848529 | 1.745542333 | 20.00885855 | 0.004300546 |
| RPS14P4 | 0.718152843 | 0.517814448 | 0.996000609 | 0.047258216 |
| OACYLP | 3.088804918 | 1.182345597 | 8.069312261 | 0.021344408 |
| CACNA2D3 | 2.710349667 | 1.431325073 | 5.132303941 | 0.002207628 |
| PDE1B | 1.439526556 | 1.134184076 | 1.827072649 | 0.002743219 |
| SPARC | 1.001414727 | 1.000129667 | 1.002701438 | 0.030937849 |
| AP000695.1 | 1.373767093 | 1.112102678 | 1.696998005 | 0.003224169 |
| BICC1 | 1.088461003 | 1.01721447 | 1.164697702 | 0.014123073 |
| NUDT10 | 1.922011593 | 1.343993407 | 2.748621046 | 0.000343875 |
| PLXDC1 | 1.226932023 | 1.065754027 | 1.412485574 | 0.004424127 |
| RNU7-45P | 0.958672659 | 0.919449057 | 0.999569536 | 0.047685051 |
| RMI1 | 0.88484926 | 0.801808018 | 0.976490874 | 0.014969897 |
| DGKI | 7.100426564 | 2.436714305 | 20.69017993 | 0.000327946 |
| PLCL1 | 1.810037186 | 1.266010436 | 2.587841712 | 0.001141177 |
| CHAF1A | 0.893307991 | 0.829788989 | 0.96168927 | 0.002717783 |
| MIR4635 | 0.877107579 | 0.782023404 | 0.98375279 | 0.025106348 |
| FEN1 | 0.96167895 | 0.931808834 | 0.992506583 | 0.015216609 |
| AC005324.5 | 0.339628846 | 0.1252947 | 0.920611589 | 0.033792071 |

Supplementary Table 2 Genes as independent risk factors affecting the prognosis of GC patients
